# Human Infection with *Rickettsia honei*, Thailand

**DOI:** 10.3201/eid1109.050011

**Published:** 2005-09

**Authors:** Ju Jiang, Vichai Sangkasuwan, Kriangkrai Lerdthusnee, Suchitra Sukwit, Thippawan Chuenchitra, Patrick J. Rozmajzl, Chirapa Eamsila, James W. Jones, Allen L. Richards

**Affiliations:** * Naval Medical Research Center, Silver Spring, Maryland, USA;; † Armed Forces Research Institute of Medical Sciences, Royal Thai Army, Bangkok, Thailand;; ‡ US Army Component, Armed Forces Research Institute of Medical Sciences, Bangkok, Thailand

**Keywords:** Spotted Fever Rickettsiosis, Thailand Tick Typhus, Rickettsia honei TT-118, Real-time Polymerase Chain Reaction, Multilocus Sequence Typing, Phylogenetic Analysis, dispatch

## Abstract

Human spotted fever rickettsiosis was detected molecularly by 2 real-time polymerase chain reaction (PCR) assays performed on DNA extracted from a Thai patient's serum sample. Sequences of PCR amplicons from 5 rickettsial genes used for multilocus sequence typing were 100% identical with those deposited with GenBank for *Rickettsia honei* TT-118.

The original Thai tick typhus isolate, TT-118, was obtained from a mixed pool of *Ixodes* sp. and *Rhipicephalus* sp. larval ticks from *Rattus rattus* trapped in Chiangmai Province, Thailand, in 1962 (*1*) and has recently been determined to be a strain of *Rickettsia honei*, the etiologic agent of Flinders Island spotted fever (*2*). No isolate has been associated with Thai tick typhus in humans, and TT-118 was found only to be moderately pathogenic for guinea pigs and gerbils (*1*). However, evidence of spotted fever rickettsiosis has been seen in Thailand; this evidence comes from 2 reports of a total of 11 cases, 3 cases from Chiangmai and 8 cases from the Thailand-Burma border. All 11 patients had signs and symptoms characteristic of spotted fever rickettsiosis, and their sera were reactive to spotted fever group (SFG) rickettsial antigens, including those derived from TT-118 (*3**,**4*). Additional proof of the presence of spotted fever rickettsiae in Thailand derives from rodent (*5*) and human (*6**,**7*) serosurveys. In addition, spotted fever agents have been demonstrated in Thai ticks by using molecular biology techniques to detect rickettsiae (*8**–**10*). Collectively, these reports indicate that SFG rickettsiae and rickettsioses exist within Thailand. However, at the time of this writing, detection of an SFG rickettsia from a human source had not been reported in Thailand.

## The Study

We describe the first detection of *R. honei* TT-118 or a very similar strain from a 36-year-old male freelance photographer living in Bangkok, Thailand, who complained of a febrile illness and was admitted to a nearby hospital on December 21, 2002 (V. Sangkasuwan et al., unpub. data). Blood was collected and submitted to the Armed Forces Research Institute of Medical Sciences, Thailand, where the serum was determined to be positive for antibodies to spotted fever rickettsiae. A portion of this serum sample, without identifiers, was subsequently sent to the Naval Medical Research Center for confirmatory serologic and molecular diagnosis. To confirm the original serologic report, the patient's serum was tested for spotted fever, typhus, and scrub typhus group-specific immunoglobulin (Ig) G by enzyme-linked immunosorbent assay using *R. rickettsii* R (ATCC VR891), *R. typhi* Wilmington whole cell antigen, and KpKtGm r56 recombinant antigen, as previously described (*11**,**12*). The patient's serum was confirmed to have IgG to SFG rickettsiae (titer >1:6,400) but not to have antibodies to *Orientia tsutsugamushi* or *R. typhi*.

To ascertain whether the patient's serum contained molecular evidence of SFG rickettsia, DNA was extracted from 150 μL of the patient's serum (DNeasy Tissue Kit, Qiagen, Valencia, CA, USA). Three micrograms Poly(dA) (Sigma Chemical Co., St. Louis, MO, USA) was added as a DNA carrier. Two real-time polymerase chain reaction (PCR) assays were performed to determine if rickettsial nucleic acid was detectable in the patient's serum sample: 1) the *Rickettsia* genus–specific real-time PCR assay amplified and detected a 115-bp segment of the 17-kDa antigen gene and 2) the rickettsial SFG-specific real-time PCR amplified and detected a 128-bp segment of the *ompB* with a SmartCycler (Cepheid, Sunnyvale, CA, USA), as previously described (*13*). Both assays demonstrated the presence of the target sequences in the serum sample with Ct values of 36.22 and 36.87, respectively.

To identify which SFG rickettsia was responsible for the patient's febrile illness, segments of 5 rickettsial genes were amplified by PCR, and the amplicons produced were sequenced and compared to reported sequences of other SFG rickettsiae. New oligonucleotide primers were selected from the conserved regions of *ompB*, *ompA*, and *sca4* after alignment of at least 19 rickettsial sequences: RompB11F (ACCATAGTAGCMAGTTTTGCAG), Rak1452R (SGTTAACTTKACCGYTTATAACTGT), RhoA1F (GAATAACATTACAAGCTGGAGGAA), RR657R (TATTTGCATCAATCSYATAAGWA), RhoA4336F (AGTTCAGGAAACGACCGTA), RrD749F (TGGTAGCATTAAAAGCTGATGG), RrD2685R (TTCAGTAGAAGATTTAGTACCAAAT), RrD1826R (TCTAAATKCTGCTGMATCAAT), and RrD1275R (TGTTAAACCTGYATTTACATAAT). All other primers used have been previously described (*2**,**14**,**15*).

To produce the amplicons used for sequencing, 2 μL or 4 μL of the sample DNA preparation was added to either a 25- or 50-μL reaction mixture, respectively, containing 0.5 μmol/L (for *ompA* and *ompB*), or 0.3 μmol/L (for 17-kDa gene, *gltA* and *sca4*) of forward and reverse primers, and PCR SuperMix High Fidelity (Invitrogen, Carlsbad, CA, USA). Two microliters of PCR products was used as template in the nested PCRs. Each PCR was performed on a TGradient Thermocycler (Whatman Biometra, Göttingen, Germany) and incubated at 94°C for 1 min followed by 40 cycles of denaturation at 94°C for 30 s; annealing at 48°C (*gltA*), 50°C (*ompA* and *ompB*), 52°C (sca4), or 58°C (17-kDa gene) for 1 min; and elongation at 70°C for 1–2 min. After the amplification steps were completed, the reaction mixtures were exposed to a final elongation step at 72°C for 7 min, and PCR products were visualized with ethidium bromide (GIBCO BRL Life Technologies, Inc., Gaithersburg, MD, USA) on 1.5% agarose gels after electrophoresis. Each mastermix for PCR was prepared in a clean room separated from where the DNA templates were added; no research with *R. honei* TT-118 had been conducted in our laboratory. A positive control DNA (*R. parkeri* genomic DNA) and a negative control (molecular biology grade water, GIBCO) were run at the same time under the same condition as the sample. The negative control consistently produced no detectable product. A 1,328-bp PCR product of *ompB* from the positive control DNA was sequenced and showed 100% identity with the published *R. parkeri ompB* sequence. The sample PCR products from the 17-kDa gene and the nested PCR products of *gltA*, *ompA*, *ompB,* and *sca4* were purified by using a QIAquick PCR purification kit (Qiagen). The BigDye Terminator v 3.0 Ready Reaction Cycle Sequencing Kit (Applied Biosystems, Foster City, CA, USA) was used in subsequent sequencing reactions, according to manufacturer's instructions. Sequencing products were purified by using Performa Gel Filtration Cartridges (EdgeBioSystems, Gaithersburg, MD, USA), and sequencing was performed on an ABI Prism 3100 Genetic Analyzer (Applied Biosystems). The primers used for PCR amplification were the same as those used for the sequencing reactions. At least 2 sequencing reactions were performed for each strand of DNA. Sequences were assembled with Sequencher 4.0 (Gene Codes Corporation Inc., Ann Arbor MI, USA), and basic local alignment search tool (BLAST) searches were managed on the NCBI Web site (http://www.ncbi.nlm.nih.gov/blast/).

A 434-bp fragment from the 17-kD antigen gene was amplified by standard PCR, and the sequence between bases 67 and 458 of the open reading frame (ORF) was determined to be 100% identical with the published sequences of *R. honei* strain TT-118 and *R. honei* strain RB, and 99.7% identical with *R. honei* strain "south Texas *A.* [*Amblyomma*] *cajennense* SFG rickettsia." Two PCR fragments were produced for *gltA* by nested PCR that included bp 82 to 1178 of the ORF. A 1069-bp sequence was obtained after assembling the sequences of the 2 fragments. BLAST search showed this sequence to be 100% identical with that of *R. honei* TT-118 and 99.9% with *R. honei* strain RB. A 741-bp *ompB* fragment was amplified by nested PCR. Sequence of this fragment was found to have 100% identity with *R. honei* TT-118 and 99.8% with *R. honei* RB. The 1,403-bp *ompA* amplicon sequence had 100% identity with *R. honei* TT-118 and *R. honei* RB *ompA* published sequences. The 1,090-bp sequence determined for *sca4* was 100% identical with that published for *R. honei* TT-118.

## Conclusions

Molecular detection of *R. honei* TT-118 in a clinical specimen from a patient suspected of having spotted fever rickettsiosis was achieved after clinical diagnosis and serologic analysis. To determine the identity of SFG agent, standard PCR and nested PCR procedures were conducted to produce amplicons from 5 rickettsial genes for multilocus sequence typing (MLST) (*10**,**15*). The 17-kDa antigen gene, a highly conserved gene among members of the genus *Rickettsia*, was used to confirm the relationship of the unknown agent to known rickettsiae. Similarly, DNA encoding *gltA* was used to ascertain the relationship of the unknown agent to other agents within the genus *Rickettsia*. The sequences of the other 3 gene segments (*ompB*, *ompA*, and *sca4*) used in the MLST scheme are much more variable among the rickettsiae and, therefore, they provided more information regarding the identity of the unknown agent.

BLAST searches against the determined sequences of segments amplified from the 17-kDa antigen, citrate synthase, OmpB, OmpA, and Sca4 genes of the unknown agent showed 100% identity with those sequences deposited within GenBank for *R. honei* TT-118. The closest other neighbors to the unknown agent included *R. honei* RB (the type strain of *R. honei*) and *R. honei* "south Texas *A. cajennense* SFG rickettsia." Thus, the patient at the time of his illness was infected with *R. honei* TT-118 or a very similar strain of SFG rickettsiae.
